# The association between physical activity and anxiety in college students: parallel mediation of life satisfaction and self-efficacy

**DOI:** 10.3389/fpubh.2024.1453892

**Published:** 2024-10-03

**Authors:** Jiaxin Deng, Yongfeng Liu, Tong Wang, Weicheng Li

**Affiliations:** School of Sports Training, Chengdu Sport University, Chengdu, China

**Keywords:** anxiety, negative emotions, self-efficacy, life satisfaction, physical activity

## Abstract

**Objective:**

To explore the functions that physical activity (PA), life satisfaction, and self-efficacy have in the process of coping with anxiety among Chinese college students, and to explore the mediating role of life satisfaction and self-efficacy in this process.

**Methods:**

Participants were 358 college students (186 males, 172 females, mean age 20.88, SD = ±1.80). Psychosocial tests including the Physical Activity Rating Scale (PARS-3), College Student Life Satisfaction Scale (CSLSS), General Self-Efficacy Scale (GSES), and Generalized Anxiety Disorder Scale (GAD-7) were completed. Correlations between variables were calculated using Pearson’s test. The mediation model was tested using the SPSS 26.0 PROCESS macro and regression bootstrap.

**Results:**

Physical activity showed a negative correlation with anxiety (*β* = −0.1617, *p < 0.001.*, life satisfaction and self-efficacy played a role in the relationship between physical activity and anxiety. More importantly, physical activity was associated with anxiety through parallel mediating effects of life satisfaction and self-efficacy, with a total mediating effect of 55.34%.

**Conclusion:**

By increasing college students’ participation in physical activity, it helps to promote the growth of their life satisfaction and self-efficacy, which is the key to reducing anxiety and promoting college students’ mental health.

## 1 Introduction

Negative emotions significantly contribute to mental disorders, with anxiety and depression being the most prevalent, ultimately becoming primary causes of the global mental health burden (1). The expression pathways of anxiety-specific symptoms vary among individuals. Anxiety, defined as the anticipation of a future threat, manifests mainly as episodic or persistent anxiety, tension, or panic, often accompanied by muscle tension, restlessness, fatigue, difficulty concentrating, irritability, and sleep disturbances (2). Studies estimate the global prevalence of anxiety disorders at 7.3% (3). Adolescence and early adulthood are critical periods for the onset of anxiety, which often emerges during childhood and adolescence and develops into disorders (4). Social factors such as societal development, increasing competition, and educational reforms have exacerbated anxiety and depression among college students. A lack of physical exercise, leading to obesity and other issues (58), further diminishes life satisfaction and self-efficacy, worsening mental health conditions (5). As society places greater emphasis on mental health, the anxiety experienced by college students is gaining increasing attention in China and worldwide. Some studies have shown that the dramatic increase of social pressure in China, such as the pressure of academics and employment, has led to the Chinese college student population being more prone to anxiety, thus affecting their mental health. How to alleviate anxiety is becoming a hot topic in Chinese academic circles (6–8). In recent years, the role of physical activity in alleviating anxiety has garnered significant scholarly attention in studies exploring the relationship between physical exercise and anxiety (9, 10).

Physical activity, defined as engaging in exercises of specific frequency, intensity, and duration, has been shown through theoretical models and empirical studies to have a protective effect on mental health. Participation in physical activity can markedly improve negative emotions caused by stress or other factors, reduce anxiety sensitivity, and consequently diminish anxiety levels (11, 12). Conversely, engaging in physical activity stimulates the production of endorphins and other substances in the body, enhancing an individual’s sense of excitement. Additionally, the socialization and collaboration involved in exercise can significantly improve self-efficacy, providing a positive experience and increasing life satisfaction, thereby effectively reducing negative emotions such as anxiety (8, 57). The (13) report by the American Sports Guide Information Council emphasizes that physical activity can alleviate anxiety (14), and evidence-based studies corroborate that it is an effective, safe, and low-cost method for anxiety relief (15). Therefore, physical activity plays a crucial role in reducing anxiety. Studies indicate that the college population faces increasing stress and anxiety, suggesting that physical activity may be an effective intervention to mitigate their anxiety levels (16).

Life satisfaction significantly influences anxiety. It is a subjective judgment about one’s quality of life, involving a comparison between an individual’s standard of living and their own expectations (17). By measuring the alignment between their current life, expected life, and actual life, it reflects a person’s psychological well-being. Recent studies have increasingly confirmed the crucial role that life satisfaction plays in predicting mental health issues. Scholars analyzing the relationship between life satisfaction and various mental disorders have found that it is associated not only with depression, suicidal tendencies, alcohol dependence, and drug addiction but also significantly correlated with anxiety disorders (18). Furthermore, life satisfaction is associated with anxiety and mood disorders, with lower life satisfaction linked to poor psychological states (19). Physical activity also influences life satisfaction, with numerous studies demonstrating that engaging in physical exercise enhances individual life satisfaction. Additionally, improving self-efficacy is recognized as an effective method for alleviating anxiety. Self-efficacy, the confidence in one’s ability to perform tasks with the skills one possesses, is influenced not only by personal abilities and skills but also by emotional state and past experiences. Physical activity has been shown to increase self-efficacy, thereby reducing anxiety (20, 21).

Many studies have examined the dangers of anxiety, the factors influencing it, and ways to alleviate it. Participation in physical exercise, improving self-efficacy, and increasing life satisfaction have been shown to effectively reduce anxiety, confirming the association between these factors (22, 23). However, there is a lack of research specifically exploring the impact of physical activity on anxiety reduction among college populations. In positive psychology, life satisfaction and self-efficacy are significant variables. Related studies highlight their role in anxiety alleviation, providing a basis for further research. Especially in the social context of China, the influence brought by different regions and nationalities might be different for college students’ anxiety (24, 25). This report aims to investigate the relationship between physical activity and anxiety among college students. It proposes a structural equation model to analyze the mediating roles of self-efficacy and life satisfaction, offering theoretical and empirical evidence to reduce anxiety in college students and improve our understanding of the psychological mechanisms involved.

## 2 Theoretical foundations and hypotheses

### 2.1 Physical activity and anxiety

Any bodily movement executed by the skeletal musculature, necessitating an expenditure of energy, encompassing both formal physical exertion and routine activities like ambulation, labor, and domestic chores, is defined as physical exercise. Drawing upon the tenets of the Emotional Effects Theory and the Social Withdrawal Theory, physical activity exhibits ameliorative and therapeutic potential for mental health afflictions such as depression and anxiety. Research has elucidated that sedentariness constitutes the primary etiological factor for numerous chronic ailments, with deleterious alterations in mood and anxiety attributed to physical inactivity (26). A study examining the correlation between daily exercise habits and anxiety levels revealed that a cohort of 1,661 adults diagnosed with anxiety disorders exhibited significant amelioration in anxiety symptoms following interventions involving physical activity; furthermore, heightened levels of physical activity corresponded to diminished anxiety levels (27). Hence, predicated upon both theoretical frameworks and empirical evidence, we posit Hypothesis 1: Physical activity exerts a negative influence on anxiety.

### 2.2 The mediating role of life satisfaction

Life satisfaction emerges as a focal point within contemporary positive psychology research. It denotes an individual’s subjective assessment of their overall quality of life, serving as a comprehensive metric for appraising one’s well-being. With the ongoing advancement of positive psychology, there is a growing recognition that enhancing life satisfaction constitutes a pivotal avenue for fostering both physical and mental health development. Within the framework of exercise psychology theory, physical activity emerges as a potent means to augment life satisfaction. Empirical investigations have revealed a link between life satisfaction and anxiety. Notably, interventions aimed at enhancing happiness, optimism, and sleep quality among older adults have been shown to elevate life satisfaction, consequently attenuating levels of depression and anxiety (28). Furthermore, a study encompassing 1,662 college students corroborated a negative correlation between life satisfaction and anxiety, with heightened life satisfaction correlating with diminished anxiety levels (29). Thus, drawing upon the findings of prior research and the prevailing phenomena, we posit Hypothesis 2: Life satisfaction may serve as a mediating factor in the relationship between physical activity and anxiety.

### 2.3 Mediating role of self-efficacy

Self-efficacy stands as a pivotal determinant shaping an individual’s perceived capacity to engage in a specific behavior prior to its execution. Previous scholarly inquiries have elucidated self-efficacy’s role in ameliorating adverse emotional states among adolescents and its correlation with life satisfaction. While fewer investigations have directly explored self-efficacy’s predictive capacity regarding life satisfaction, Moksnes’ study unveiled a noteworthy impact of self-efficacy on adolescents’ life satisfaction (30). Furthermore, evidence suggests that engagement in physical activity not only directly enhances life satisfaction but also augments it indirectly by bolstering individuals’ self-efficacy and mental resilience. This underscores the mediating function of self-efficacy in this process (31). Consequently, we posit Hypothesis 3: Self-efficacy may serve as a mediating factor in the relationship between physical activity and anxiety.

### 2.4 The effect of gender in the relationship between physical activity and anxiety

The mediating variable has been employed to elucidate the impact of physical activity on anxiety, yet it may lack sufficient explanatory power regarding the variation of this effect across different contexts. Consequently, drawing upon social role theory and other pertinent empirical studies, we introduced gender as a moderating variable to more effectively explore the effects of physical activity on anxiety in diverse scenarios. According to the social theory of gender roles, individuals are ascribed different roles and responsibilities through socialization, resulting in gender differences in their future development (32). Furthermore, boys and girls exhibit varying levels of anxiety when confronted with challenges or problems in school life, with some studies indicating that boys experience significantly lower levels of anxiety compared to girls (33). At the university level, students demonstrate different degrees of socialization, and research has confirmed that girls, and not just boys, are more prone to anxiety and more likely to express it (34). Additionally, male students are more inclined than female students to engage in physical activity at university, potentially making it easier for them to alleviate anxiety through such activities. Conversely, female students might receive some level of support from their peers. Ultimately, synthesizing previous studies and grounded in social role theory, this study incorporated gender as a moderating variable in constructing a parallel mediation model to investigate the effects of physical activity on anxiety and its mechanisms. Specifically, this study first considered the mediating roles of life satisfaction and self-efficacy in the relationship between physical activity and anxiety, then examined the moderating role of gender on these mediating pathways, aiming to provide empirical support and theoretical guidance for college students’ participation in physical activity to mitigate anxiety.

## 3 Materials and methods

### 3.1 Participants

Drawing upon prior research and considering the disparities in experimental and variable designs from this study (35), alongside the estimation of the ex-ante sample size utilizing G-power software, with parameters set as *f* = 0.25, *α =* 0.05, and power (1-*β*) = 0.95, the requisite sample size for this investigation was determined to be 210 individuals (36). Subsequently, employing a randomized convenience sampling technique, 400 school personnel hailing from two universities in Chengdu City, Sichuan Province, situated in central China, were enlisted for participation in this study. Recruitment was conducted through face-to-face offline classroom interactions and online questionnaire dissemination. Prospective participants were briefed on the study’s objectives and given the opportunity to express their interest in participation. Upon providing written informed consent, participants were tasked with completing a comprehensive questionnaire aimed at gathering pertinent demographic data and facilitating subsequent follow-up procedures. The questionnaire distribution and collection spanned from March 2024 to May 2024. Subsequently, after identifying and excluding 42 invalid questionnaires characterized by rapid completion, irregular responses, and logical inconsistencies, the final sample comprised validly completed questionnaires from 358 participants (186 males, and 172 females), with a mean age of 20.88 years and a standard deviation of ±1.80. The questionnaire achieved an effective return rate of 85.9%. Details regarding survey participation are delineated in Table 1. It is hereby affirmed that this study adhered to the principles outlined in the 1964 Declaration of Helsinki and its subsequent revisions. Additionally, we attest that the research methodologies employed received approval from the Ethics Committee of the School of Sports Training, Chengdu Institute of Physical Education and Sports (CT), approval no. CTYLL2024001, and that informed consent was obtained from all participating individuals.

**Table 1:** Descriptive statistics (*n* = 358).

Projects	Categories	Cases	Percentage
Gender	Male	186	51.96%
	Female	172	48.04%
Age	19	70	19.55%
	20	93	25.98%
	21	108	30.17%
	22	56	15.64%
	>23	31	8.66%
student’s degree	Undergraduate	302	84.36%
	Bachelor’s degree	48	13.41%
	PhD	8	2.23%

### 3.2 Measurement instruments

#### 3.2.1 Physical Activity Rating Scale (PARS-3)

In this study, we employed the Physical Activity Rating Scale, revised by Leung (37). This scale evaluates participants’ physical activity levels based on the intensity, frequency, and duration of their physical activity engagement, categorizing individual activity levels across five tiers, assessed on a scale from 1 to 5. The total physical activity score is calculated as follows: exercise intensity × (exercise duration − 1) × exercise frequency, with scores ranging from 0 to 100 points. A higher score corresponds to a greater level of physical activity, with scores ≤19 indicating minimal activity, scores between 20 and 42 indicative of moderate activity, and scores ≥43 suggestive of substantial activity. In this study, the Cronbach’s *α* coefficient for this scale was calculated to be 0.816.

#### 3.2.2 College Student Life Satisfaction Scale (CSLSS)

In this study, we utilized the College Students’ Life Satisfaction Scale (CSLSS), revised by Wang and Shih (38). This scale comprises six sections, encompassing objective satisfaction items (e.g., academic performance, social relationships, etc.) and subjective satisfaction items (e.g., overall life satisfaction, etc.). Responses are provided on a 5-point Likert scale, ranging from “very satisfied” to “very dissatisfied.” The CSLSS exhibits strong internal consistency, as evidenced by its overall Cronbach’s *α* coefficient of 0.894.

#### 3.2.3 General Self-Efficacy Scale (GSES)

In this study, we employed the General Self-Efficacy Scale (GSES), developed by Schwarzer et al. (39). This scale comprises 10 items rated on a 5-point Likert scale, ranging from “very satisfied” to “very dissatisfied.” A higher total score signifies greater self-efficacy, while lower scores denote lower self-efficacy levels. The GSES demonstrated high internal consistency in this study, as indicated by its overall Cronbach’s *α* coefficient of 0.959.

#### 3.2.4 Generalized Anxiety Disorder Scale (GAD-7)

For this study, the Generalized Anxiety Disorder Scale (GAD-7) developed by Spitzer et al. (40) was selected. This self-report questionnaire assesses the severity of generalized anxiety disorder symptoms. Chinese scholars have translated and tested the reliability of the scale, demonstrating good internal consistency within the Chinese population (41). Participants were queried about the frequency of experiencing seven symptoms of generalized anxiety disorder over the past 2 weeks. Responses were rated on a 4-point scale ranging from “not at all” to “almost every day,” with total scores ranging from 0 to 21. Cut-off values of 5, 10, and 15 were established for mild, moderate, and severe anxiety, respectively. The GAD-7 exhibited high internal consistency in this study, with a Cronbach’s *α* coefficient of 0.953.

### 3.3 Statistical analysis

**Figure 1:**
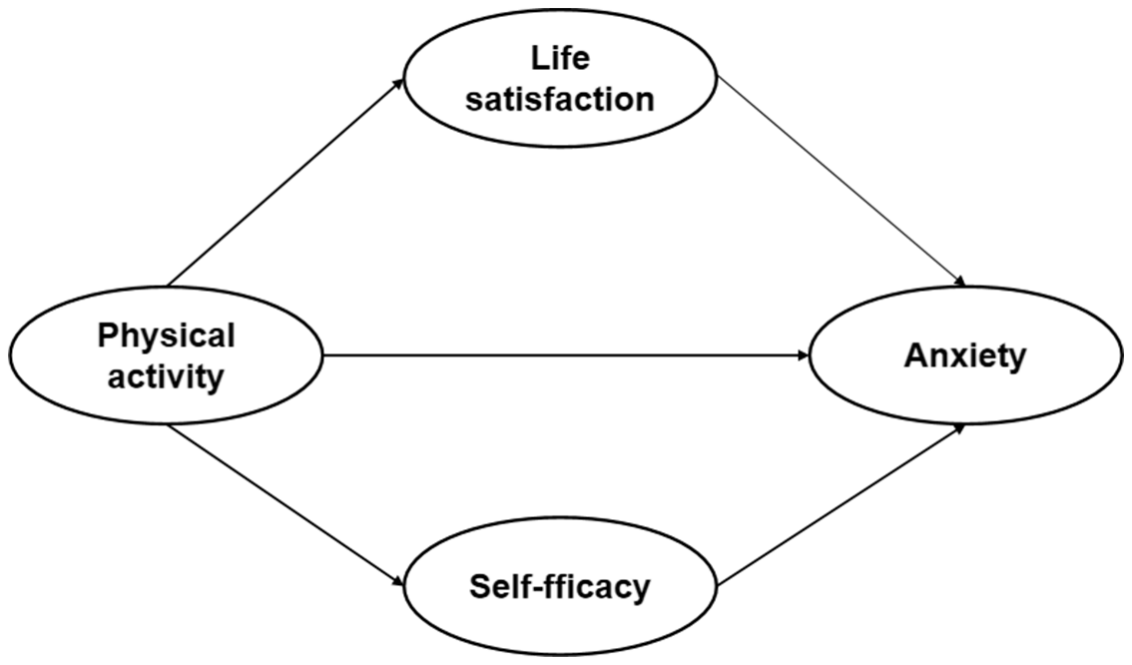
Hypothetical model of this study.

Data analysis was conducted using the SPSS software package (version 26.0). Categorical variables were represented as frequencies (n) and percentages (%). Gender was analyzed using analysis of covariance (ANOVA), independent samples, and rank sum ratio tests. The Kolmogorov test and normal distribution test were employed to assess differences in physical activity, life satisfaction, self-efficacy, and anxiety. Cohen’s or Pearson’s r correlation coefficients were computed for parametric and non-parametric tests, respectively. To explore the potential mediating roles of life satisfaction and self-efficacy between physical activity and anxiety, Model 4 in the SPSS PROCESS 4.0 plug-in developed by Hayes was utilized for analysis (42). In this model, physical activity served as the independent variable, while life satisfaction and self-efficacy were considered as mediating variables, and anxiety served as the dependent variable (see Figure 1). Additionally, age and gender were included as covariates to investigate their impact on this process. This analysis facilitated the prediction of the mediating effects of life satisfaction and self-efficacy in the relationship between physical activity and anxiety. The mediation hypothesis was tested using the bias-corrected bootstrap method on a sample of 5,000, with confidence intervals (95%) calculated. Significance was considered when the confidence interval did not exceed zero.

## 4 Results

### 4.1 Common method bias

The relevant data underwent the Harman one-way test, the KMO test, and Bartlett’s sphericity test. The KMO value was 0.966, and Bartlett’s value was 9372.716 with 703 degrees of freedom, yielding a *p*-value <0.001. These results suggest that the data are conducive to factor analysis, with all variables rotated. Exploratory factor analysis revealed four factors with eigenvalues exceeding 1, collectively explaining a maximum factor variance of 39.62% (<40%). Consequently, these findings indicate the absence of common method bias in the study. To assess potential multicollinearity among variables, anxiety was standardized as the dependent variable, while physical activity, life satisfaction, and self-efficacy served as independent variables for covariate diagnosis. The tolerance values for each variable (0.773, 0.763, and 0.776) exceeded 0.1, and the variance inflation factor (VIF) values (1.293, 1.311, and 1.288) were <5. Thus, it can be inferred that the data in this study did not exhibit multicollinearity issues, rendering them suitable for further mediation effect testing.

### 4.2 Correlation analysis of physical activity, life satisfaction, self-efficacy and anxiety

Based on the findings presented in Table 2, physical activity demonstrates a significant positive correlation with both life satisfaction and self-efficacy. Conversely, anxiety exhibits a significant negative correlation with physical activity, life satisfaction, and self-efficacy. These correlations suggest meaningful associations among the variables, implying that life satisfaction and self-efficacy may play influential roles in the relationship between physical activity and anxiety. This provides a rationale for conducting further tests to explore their mediating effects. As for the model fit indices, chi-square values, root mean square error of approximation (RMSEA) values, and comparative fit indices (CFI) were reported (*df* = 1.095, RMSEA = 0.016, CFI = 0.993, and GFI = 0.807), indicating a good fit of the model and suitability for additional mediation analysis.

**Table 2:** Correlation analysis of physical activity, life satisfaction, self-efficacy and anxiety among college students (*N* = 358).

Variables	Mean (*M*)	Standard deviation (*SD*)	1	2	3	4
Physical activity	24.80	9.85	1			
Life satisfaction	3.55	1.05	0.408^**^	1		
Self-efficacy	3.56	1.02	0.389^**^	0.404^**^	1	
Anxiety	40.38	11.33	−0.389^**^	−0.440^**^	−0.458^**^	1

** *p < 0.01.*

### 4.3 Parallel mediation analysis of subjective well-being and self-efficacy

The analysis of mediated effects was conducted through bootstrap procedures employing Model 4 within Process 4.0, an SPSS macro add-in crafted by Hayes. This model was specifically tailored to examine the mediated effects, employing gender and age as covariates. The independent variable under scrutiny was physical activity, while life satisfaction and self-efficacy served as mediators, with anxiety as the dependent variable and gender and age as covariates. Within this investigation, the sample replication consisted of 5,000 cases, and the standard confidence interval was set at 95% (43). The outcomes of the regression analysis are delineated in Table 3. Initially, physical activity exhibited a direct positive association with both life satisfaction and self-efficacy. Furthermore, a negative correlation between physical activity and anxiety was observed (*β* = −0.1617, *p < 0.001.*, thus confirming Hypothesis 1: physical activity may exert a negative impact on anxiety. Subsequently, the findings indicate that life satisfaction demonstrated an inverse relationship with anxiety (*β* = −0.2476, *p < 0.001.*, and similarly, self-efficacy exhibited a negative association with anxiety (*β* = −0.2856, *p < 0.001.*.

**Table 3:** Regression analysis of the parallel mediation model of physical activity on anxiety (*N* = 358).

		Overall fit index				Significance of regression coefficients
Outcome Variables	Predictor variables	*R*	*R* ^2^	*F*	*β*	*t*
Life satisfaction	Physical activity	0.4076	0.1662	70.943	0.3865	8.4228^***^
Self-efficacy	Physical activity	0.3895	0.1517	63.6624	0.3667	7.9789^***^
	Life satisfaction					
Anxiety	Physical activity	0.5576	0.3109	53.2311	−0.1617	−3.4614^***^
	Life satisfaction				−0.2476	−4.9928^***^
	Self-efficacy				−0.2856	−5.7680^***^

*** *p < 0.001.*

Table 4 reveals that 55.34% of the overall mediated effect in the mediation analysis, with a 95% confidence interval CI [−0.2621, −0.1419], does not encompass 0, suggesting that the parallel mediation model incorporating life satisfaction and physical activity as mediating variables is upheld. Moreover, three paths of influence are evident in the impact of physical activity on anxiety. These paths include Path 1: the direct effect of physical activity on anxiety (*β* = −0.1617); Path 2: the mediating effect of life satisfaction between physical activity and anxiety (*β* = −0.0957, accounting for 26.43% of the effect share); and Path 3: the mediating effect of self-efficacy between physical activity and anxiety (*β* = −0.1047, constituting 28.91% of the effect share). The 95% confidence intervals for these three paths do not include zero, signifying that physical activity, life satisfaction, and self-efficacy can individually influence anxiety. Concurrently, life satisfaction and self-efficacy serve as parallel mediators in the relationship between physical activity and college students’ anxiety, thus corroborating Hypothesis H2: life satisfaction mediates the relationship between physical activity and anxiety, and Hypothesis H3: self-efficacy mediates the relationship between physical activity and anxiety.

**Table 4:** Parallel mediation tests of life satisfaction and self-efficacy on physical activity and anxiety (*N* = 358).

	Effect	Boot SE	Boot LLCI	Boot ULCI	Proportion of effect
Aggregate effect	−0.3621	0.0455	−0.4515	−0.2727	
Total direct effect	−0.1617	0.0467	−0.2535	−0.0698	45.66%
Total indirect effect	−0.2004	0.0307	−0.2621	−0.1419	55.34%
Path 1: physical activity → life satisfaction → anxiety	−0.0957	0.0232	−0.1437	−0.0524	26.43%
Path 2: physical activity → self-efficacy → anxiety	−0.1047	0.0222	−0.1501	−0.0634	28.91%
Contrasting indirect effects (1–2)	0.009	0.0335	−0.0582	0.0747	

### 4.4 Moderating effect of gender

Subsequently, a moderated mediation analysis was conducted using PROCESS Model 7. The results revealed that the interaction between physical activity and gender in predicting anxiety was not significant after controlling for grade level and education. Further analysis of the mediation effects under different moderated variables also yielded no significant results. Therefore, the findings indicated that there was no significant moderating effect, suggesting no substantial gender difference in the mediating effects of life satisfaction and self-efficacy.

In summary, this study verified the inhibitory effect of physical activity on college students’ anxiety, in which life satisfaction and self-efficacy played a mediating effect respectively, but gender did not produce a significant moderating effect.

## 5 Discussion

**Figure 2:**
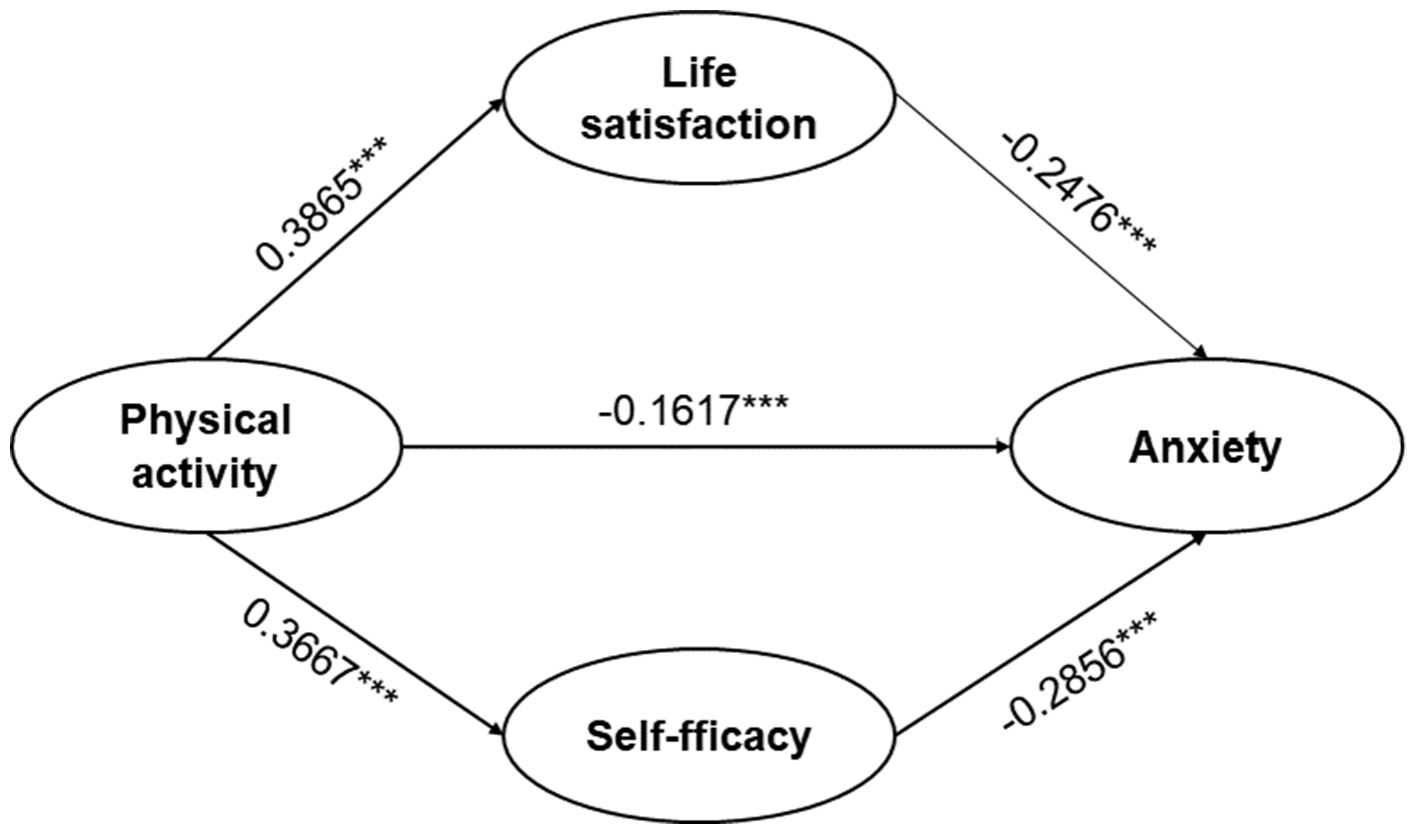
Parallel mediation model of life satisfaction and self-efficacy in physical activity and anxiety. ^***^p < 0.001.

The study explored the impact of life satisfaction and self-efficacy on anxiety among college students in western China, as well as the association between physical activity and college students’ anxiety, thereby confirming hypotheses 1, 2, and 3. Furthermore, the study elucidated that college students enhance their life satisfaction and self-efficacy through engagement in physical activity, consequently mitigating their anxiety. This finding offers valuable insights for the development of future mental health intervention programs aimed at alleviating anxiety among college students (see Figure 2).

### 5.1 Direct effect of physical activity on anxiety

The findings of this study indicate that college students who engage in physical activity experience a reduction in anxiety levels, thus validating Hypothesis 1. This outcome aligns with Herring’s research (44), further corroborating the anxiety-relieving effects of physical activity. The negative predictive impact of physical activity on college students’ anxiety remained significant even after accounting for mediating variables. Physical activity holds a crucial role in the daily lives of college students, offering opportunities for enhancing physical health, subjective well-being, and social engagement. Conversely, insufficient physical activity stands as a significant contributor to chronic diseases. In accordance with the neural hypothesis, sustained and regular physical activity is postulated to mitigate the prevalence of negative emotions by enhancing the expression of neurotrophic factors in the brain and diminishing the stress response (45). Concurrently, according to the self-determination theory, individuals’ persistent commitment to physical activity, attainment of exercise objectives, and overcoming associated challenges can serve as psychological buffers, fostering positive emotional experiences (46). Drawing upon the outcomes of this study and prior research, it is inferred that physical exercise can effectively alleviate anxiety and foster the enhancement of college students’ psychological well-being.

### 5.2 Mediating role of life satisfaction

The findings of this study reveal a robust correlation between life satisfaction, physical activity, and anxiety. Path analysis elucidates that life satisfaction exerts a significant mediating influence on the relationship between physical activity and anxiety. It is established that life satisfaction is inversely associated with anxiety, wherein heightened life satisfaction corresponds to lower levels of anxiety (43). Individuals with elevated life satisfaction tend to embrace a more active lifestyle and adopt positive health behaviors and coping mechanisms when confronted with mental health challenges such as anxiety (47). Meanwhile, significant disparities in life satisfaction and anxiety levels during physical exercise were observed among college students with varying educational backgrounds, grades, and genders. Additionally, our study found that physical activity can effectively enhance individual life satisfaction and diminish anxiety levels by elevating the level of life satisfaction. Furthermore, some research has explored the capacity of physical activity to bolster individuals’ mental resilience, thereby augmenting their life satisfaction. Certain studies have indicated a correlation between an individual’s life satisfaction and their overall quality of life, with heightened life satisfaction facilitating more effective coping with negative emotions, thus enhancing overall quality of life (48). The present study investigated the role of physical activity in mitigating anxiety by bolstering life satisfaction among college students. Maintaining healthy physical activity habits among this demographic can effectively heighten their life satisfaction and alleviate anxiety levels.

### 5.3 Mediating role of self-efficacy

The study findings indicated a robust correlation between self-efficacy, physical activity, and anxiety. Path analysis utilizing self-efficacy as a mediation model demonstrated that self-efficacy mediated the association between physical activity and anxiety, exerting a significant impact. As per the mediation effect analysis, physical activity not only inversely predicts anxiety among college students but also indirectly influences anxiety levels by bolstering self-efficacy, thus confirming Hypothesis H3. Furthermore, symptoms of anxiety partially elucidate the reciprocal relationship between physical activity and self-efficacy, with the magnitude of this correlation suggesting that heightened anxiety levels may stem from insufficient physical activity and diminished self-efficacy. According to the resource depletion theory, college students exposed to higher daily stressors experience greater emotional and physical depletion, thereby impacting their self-efficacy and engagement in physical activity to varying extents (49). Low self-efficacy can precipitate negative emotions such as low self-esteem and timidity among college students, consequently elevating anxiety levels (50). Given the inherent connection and mediating role of self-efficacy between physical activity and anxiety, short-term moderate-to high-intensity physical activity can yield immediate mood enhancements (51). This prompt mood elevation can positively influence an individual’s self-efficacy, thereby alleviating anxiety among college students.

### 5.4 Parallel mediating role of life satisfaction and self-efficacy

The preceding studies demonstrate a robust correlation between physical activity and anxiety. Furthermore, anxiety levels are influenced by life satisfaction and self-efficacy, prompting further investigation into the relationship among these four variables. Parallel mediation modeling between physical activity and anxiety, incorporating life satisfaction and self-efficacy as mediator variables, revealed that these factors not only serve as individual mediators but also concurrently assume parallel mediating roles in the model presentation. Notably, prior research has not reported the capacity for these factors to concurrently serve as parallel mediators. Behaviorists posit that anxiety stems from reinforcement and conditioning (52, 53). Enhancing self-efficacy can aid college students in surmounting the fear of the unknown (54), while physical exercise not only enhances college students’ self-efficacy but also fosters their life satisfaction, thereby potentially diminishing the manifestation of anxiety symptoms (55). Cognitivism contends that anxiety arises from the tendency of individuals to overestimate the peril of a situation and underestimate their ability to cope with it (56). In essence, higher levels of self-efficacy and life satisfaction prompt individuals to adopt positive and adaptive responses to future events, thereby contributing to anxiety reduction. Physical activity emerges as one of the most effective means of fostering these phenomena.

## 6 Research limitations and future perspectives

The principal contribution of this study lies in further elucidating the mechanism underlying the relationship between physical activity and anxiety among Chinese college students. It sheds light on the pivotal roles of life satisfaction and self-efficacy in this relationship, thereby enriching the body of research on the correlation between participation in physical activity and anxiety within the college student demographic. However, there are certain limitations to this study.

Firstly, its cross-sectional design restricts the ability to infer causality between physical activity and anxiety. Future scholars could validate the mediating roles of life satisfaction and self-efficacy through longitudinal studies and consider the diversity among college students in terms of majors and places of residence.

Secondly, due to data constraints, we selected life satisfaction and self-efficacy as mediating variables, potentially leading to biased findings. Subsequent researchers could corroborate the outcomes of this study by conducting scale tests on life satisfaction and self-efficacy.

Lastly, our study solely examined two mediating variables. There may exist additional mediating variables between physical activity and anxiety, prompting future investigations to incorporate more specific mediating factors such as subjective well-being and lifestyle.

## 7 Conclusion

The findings of this study demonstrate that physical exercise exerts both a direct and an indirect influence on college students’ anxiety. Indirectly, it impacts anxiety symptoms through its effects on life satisfaction and self-efficacy. Concurrently, physical exercise enhances college students’ life satisfaction and self-efficacy, thereby mitigating anxiety symptoms. In today’s era of prioritizing individual health, these findings serve as a reminder to society and educational institutions to prioritize the physical well-being of college students. In addition, the present study further demonstrates the different sources of anxiety among university students in the Chinese social context, which leads to different ways of influencing anxiety, further providing Chinese evidence for the field. Allocating opportunities for participation in physical exercise can contribute to enhancing the mental health of college students to a considerable extent.

## Data Availability

The raw data supporting the conclusions of this article will be made available by the authors, without undue reservation.
